# Assessment of variation in immunosuppressive pathway genes reveals TGFBR2 to be associated with risk of clear cell ovarian cancer

**DOI:** 10.18632/oncotarget.10215

**Published:** 2016-06-21

**Authors:** Shalaka S. Hampras, Lara E. Sucheston-Campbell, Rikki Cannioto, Jenny Chang-Claude, Francesmary Modugno, Thilo Dörk, Peter Hillemanns, Leah Preus, Keith L. Knutson, Paul K. Wallace, Chi-Chen Hong, Grace Friel, Warren Davis, Mary Nesline, Celeste L. Pearce, Linda E. Kelemen, Marc T. Goodman, Elisa V. Bandera, Kathryn L. Terry, Nils Schoof, Kevin H. Eng, Alyssa Clay, Prashant K. Singh, Janine M. Joseph, Katja K.H. Aben, Hoda Anton-Culver, Natalia Antonenkova, Helen Baker, Yukie Bean, Matthias W. Beckmann, Maria Bisogna, Line Bjorge, Natalia Bogdanova, Louise A. Brinton, Angela Brooks-Wilson, Fiona Bruinsma, Ralf Butzow, Ian G. Campbell, Karen Carty, Linda S. Cook, Daniel W. Cramer, Cezary Cybulski, Agnieszka Dansonka-Mieszkowska, Joe Dennis, Evelyn Despierre, Ed Dicks, Jennifer A. Doherty, Andreas du Bois, Matthias Dürst, Doug Easton, Diana Eccles, Robert P. Edwards, Arif B. Ekici, Peter A. Fasching, Brooke L. Fridley, Yu-Tang Gao, Aleksandra Gentry-Maharaj, Graham G. Giles, Rosalind Glasspool, Jacek Gronwald, Patricia Harrington, Philipp Harter, Hanis Nazihah Hasmad, Alexander Hein, Florian Heitz, Michelle A.T. Hildebrandt, Claus Hogdall, Estrid Hogdall, Satoyo Hosono, Edwin S. Iversen, Anna Jakubowska, Allan Jensen, Bu-Tian Ji, Beth Y. Karlan, Melissa Kellar, Joseph L. Kelley, Lambertus A. Kiemeney, Rüdiger Klapdor, Nonna Kolomeyevskaya, Camilla Krakstad, Susanne K. Kjaer, Bridget Kruszka, Jolanta Kupryjanczyk, Diether Lambrechts, Sandrina Lambrechts, Nhu D. Le, Alice W. Lee, Shashikant Lele, Arto Leminen, Jenny Lester, Douglas A. Levine, Dong Liang, Jolanta Lissowska, Song Liu, Karen Lu, Jan Lubinski, Lene Lundvall, Leon F.A.G. Massuger, Keitaro Matsuo, Valeria McGuire, John R. McLaughlin, Ian McNeish, Usha Menon, Joanna Moes-Sosnowska, Steven A. Narod, Lotte Nedergaard, Heli Nevanlinna, Stefan Nickels, Sara H. Olson, Irene Orlow, Rachel Palmieri Weber, James Paul, Tanja Pejovic, Liisa M. Pelttari, Barbara Perkins, Jenny Permuth-Wey, Malcolm C. Pike, Joanna Plisiecka-Halasa, Elizabeth M. Poole, Harvey A. Risch, Mary Anne Rossing, Joseph H. Rothstein, Anja Rudolph, Ingo B. Runnebaum, Iwona K. Rzepecka, Helga B. Salvesen, Eva Schernhammer, Kristina Schmitt, Ira Schwaab, Xiao-Ou Shu, Yurii B Shvetsov, Nadeem Siddiqui, Weiva Sieh, Honglin Song, Melissa C. Southey, Ingvild L. Tangen, Soo-Hwang Teo, Pamela J. Thompson, Agnieszka Timorek, Ya-Yu Tsai, Shelley S. Tworoger, Jonathan Tyrer, Anna M. van Altena, Ignace Vergote, Robert A. Vierkant, Christine Walsh, Shan Wang-Gohrke, Nicolas Wentzensen, Alice S. Whittemore, Kristine G. Wicklund, Lynne R. Wilkens, Anna H. Wu, Xifeng Wu, Yin-Ling Woo, Hannah Yang, Wei Zheng, Argyrios Ziogas, Simon A. Gayther, Susan J. Ramus, Thomas A. Sellers, Joellen M. Schildkraut, Catherine M. Phelan, Andrew Berchuck, Georgia Chenevix-Trench, Julie M. Cunningham, Paul P. Pharoah, Roberta B. Ness, Kunle Odunsi, Ellen L. Goode, Kirsten B. Moysich

**Affiliations:** ^1^ Department of Cancer Epidemiology, Moffitt Cancer Center, Tampa, Florida, USA; ^2^ College of Pharmacy, The Ohio State University, Columbus, Ohio, USA; ^3^ Department of Veterinary Biosciences, College of Veterinary Medicine, The Ohio State University, Columbus, Ohio, USA; ^4^ Department of Cancer Prevention and Control, Roswell Park Cancer Institute, Buffalo, New York, USA; ^5^ German Cancer Research Center (DKFZ), Division of Cancer Epidemiology, Heidelberg, Germany; ^6^ Department of Epidemiology and Department of Obstetrics, Gynecology and Reproductive Sciences, University of Pittsburgh, Pittsburgh, Pennsylvania, USA; ^7^ Women's Cancer Research Program, Magee-Women's Research Institute and University of Pittsburgh Cancer Institute, Pittsburgh, Pennsylvania, USA; ^8^ Gynaecology Research Unit, Hannover Medical School, Hannover, Germany; ^9^ Clinics of Obstetrics and Gynaecology, Hannover Medical School, Hannover, Germany; ^10^ Department of Immunology, Mayo Clinic, Rochester, Minnesota, USA; ^11^ Department of Flow & Image Cytometry, Roswell Park Cancer Institute, Buffalo, New York, USA; ^12^ Center for Personalized Medicine, Roswell Park Cancer Institute, Buffalo, New York, USA; ^13^ Department of Preventive Medicine, Keck School of Medicine, University of Southern California Norris Comprehensive Cancer Center, Los Angeles, California, USA; ^14^ Alberta Health Services-Cancer Care, Department of Population Health Research, Calgary, Alberta, Canada; ^15^ Cancer Prevention and Control, Samuel Oschin Comprehensive Cancer Institute, Cedars-Sinai Medical Center, Los Angeles, California, USA; ^16^ Cancer Prevention and Control, Rutgers Cancer Institute of New Jersey, New Brunswick, New Jersey, USA; ^17^ Obstetrics and Gynecology Center, Brigham and Women's Hospital and Harvard Medical School, Boston, Massachusetts, USA; ^18^ Department of Medical Epidemiology and Biostatistics, Karolinska Institutet, Stockholm, Sweden; ^19^ Department of Biostatistics & Bioinformatics, Roswell Park Cancer Institute, Buffalo, New York, USA; ^20^ Department for Health Evidence, Radboud University Medical Centre, Nijmegen, The Netherlands; ^21^ Department of Epidemiology and School of Medicine, University of California Irvine, Irvine, California, USA; ^22^ Byelorussian Institute for Oncology and Medical Radiology Aleksandrov N.N., Minsk, Belarus; ^23^ Department of Oncology, University of Cambridge, Strangeways Research Laboratory, Cambridge, UK; ^24^ Department of Obstetrics & Gynecology and Knight Cancer Institute, Oregon Health & Science University, Portland, Oregon, USA; ^25^ Department of Gynecology and Obstetrics, University Hospital Erlangen, Friedrich-Alexander-University Erlangen-Nuremberg, Erlangen, Germany; ^26^ Gynecology Service, Department of Surgery, Memorial Sloan Kettering Cancer Center, New York, New York, USA; ^27^ Department of Gynecology and Obstetrics, Haukeland University Hospital, Bergen, Norway; ^28^ Division of Cancer Epidemiology and Genetics, National Cancer Institute, Bethesda, Maryland, USA; ^29^ Canada's Michael Smith Genome Sciences Centre, BC Cancer Agency, Vancouver, British Columbia, Canada; ^30^ Cancer Epidemiology Centre, Cancer Council Victoria, Melbourne, Australia; ^31^ Department of Obstetrics and Gynecology, University of Helsinki and Helsinki University Central Hospital, Helsinki, Finland; ^32^ Cancer Genetics Laboratory, Research Division, Peter MacCallum Cancer Centre, St Andrews Place, East Melbourne, Australia; ^33^ Cancer Research UK Clinical Trials Unit, The Beatson West of Scotland Cancer Centre, University of Glasgow, Glasgow, UK; ^34^ Division of Epidemiology and Biostatistics, Department of Internal Medicine, University of New Mexico, Albuquerque, New Mexico, USA; ^35^ International Hereditary Cancer Center, Department of Genetics and Pathology, Clinic of Opthalmology, Pomeranian Medical University, Szczecin, Poland; ^36^ Department of Pathology and Labolatory Diagnostic, The Maria Sklodowska-Curie Memorial Cancer Center and Institute of Oncology, Warsaw, Poland; ^37^ Division of Gynecological Oncology, Department of Oncology, University Hospitals Leuven, Belgium; ^38^ Department of Community and Family Medicine, Section of Biostatistics & Epidemiology, The Geisel School of Medicine at Dartmouth, Hanover, New Hampshire, USA; ^39^ Department of Gynecology and Gynecologic Oncology, Kliniken Essen-Mitte/Evang. Huyssens-Stiftung/Knappschaft GmbH, Essen, Germany; ^40^ Department of Gynecology, Jena University Hospital - Friedrich Schiller University, Jena, Germany; ^41^ Centre for Cancer Genetic Epidemiology, Department of Public Health and Primary Care, University of Cambridge, Cambridge, UK; ^42^ Wessex Clinical Genetics Service, Princess Anne Hospital, Southampton, UK; ^43^ Department of Obstetrics, Gynecology & Reproductive Sciences and Ovarian Cancer Center of Excellence, University of Pittsburgh, Pittsburgh, Pennsylvania, USA; ^44^ Institute of Human Genetics, University Hospital Erlangen, Friedrich-Alexander-University Erlangen-Nuremberg, Erlangen, Germany; ^45^ Department of Medicine, Division of Hematology and Oncology, University of California at Los Angeles, Los Angeles, California, USA; ^46^ Department of Biostatistics, University of Kansas Medical Center, Kansas City, Kansas, USA; ^47^ Shanghai Cancer Institute, Shanghai, China; ^48^ Institute for Women's Health, Population Health Sciences, University College - London, London, United Kingdom; ^49^ Centre for Epidemiology and Biostatistics, Melbourne School of Population and Global Health, The University of Melbourne, Victoria, Australia; ^50^ International Hereditary Cancer Center, Department of Genetics and Pathology, Pomeranian Medical University, Szczecin, Poland; ^51^ Cancer Research Initiatives Foundation, Sime Darby Medical Center, Subang Jaya, Malaysia; ^52^ Department of Epidemiology, The University of Texas MD Anderson Cancer Center, Houston, Texas, USA; ^53^ Department of Gynaecology, Rigshospitalet, University of Copenhagen, Copenhagen, Denmark; ^54^ Institute of Cancer Epidemiology, Danish Cancer Society, Copenhagen, Denmark; ^55^ Division of Epidemiology and Prevention, Aichi Cancer Center Research Institute, Nagoya, Aichi, Japan; ^56^ Department of Statistical Science, Duke University, Durham, North Carolina, USA; ^57^ Department of Virus, Lifestyle and Genes, Danish Cancer Society Research Center, Copenhagen, Denmark; ^58^ Women's Cancer Program at the Samuel Oschin Comprehensive Cancer Institute, Cedars-Sinai Medical Center, Los Angeles, California, USA; ^59^ Department of Obstetrics, Gynecology and Reproductive Sciences, University of Pittsburgh School of Medicine, Pittsburgh, Pennsylvania, USA; ^60^ Division of Gynecologic Oncology, Roswell Park Cancer Institute, Buffalo, New York, USA; ^61^ Vesalius Research Center, VIB, Leuven, Belgium; ^62^ Laboratory for Translational Genetics, Department of Oncology, University of Leuven, Belgium; ^63^ Cancer Control Research, BC Cancer Agency, Vancouver, British Columbia, Canada; ^64^ College of Pharmacy and Health Sciences, Texas Southern University, Houston, Texas, USA; ^65^ Department of Cancer Epidemiology and Prevention, M. Sklodowska-Curie Memorial Cancer Center and Institute of Oncology, Warsaw, Poland; ^66^ Department of Gynecologic Oncology, The University of Texas MD Anderson Cancer Center, Houston, Texas, USA; ^67^ Department of Gynaecology, Radboud University Nijmegen Medical Centre, Nijmegen, The Netherlands; ^68^ Department of Health Research and Policy - Epidemiology, Stanford University School of Medicine, Stanford, California, USA; ^69^ Prosserman Centre for Health Research, Lunenfeld-Tanenbaum Research Institute, Mount Sinai Hospital, Toronto, Ontario, Canada; ^70^ Institute of Cancer Sciences, University of Glasgow, Glasgow, UK; ^71^ Women's Cancer, UCL EGA Institute for Women's Health, London, UK; ^72^ Women's College Research Institute, Toronto, Ontario, Canada; ^73^ Department of Pathology, Rigshospitalet, University of Copenhagen, Denmark; ^74^ Department of Epidemiology and Biostatistics, Memorial Sloan-Kettering Cancer Center, New York, New York, USA; ^75^ Department of Community and Family Medicine, Duke University Medical Center, Durham, North Carolina, USA; ^76^ Channing Division of Network Medicine, Brigham and Women's Hospital and Harvard Medical School, Boston, Massachusetts, USA; ^77^ Department of Chronic Disease Epidemiology, Yale School of Public Health, New Haven, Connecticut, USA; ^78^ Program in Epidemiology, Fred Hutchinson Cancer Research Center, Seattle, Washington, USA; ^79^ Institut für Humangenetik Wiesbaden, Wiesbaden, Germany; ^80^ Vanderbilt Epidemiology Center, Vanderbilt University School of Medicine, Nashville, Tennessee, USA; ^81^ Cancer Epidemiology Program, University of Hawaii Cancer Center, Hawaii, USA; ^82^ Department of Gynaecological Oncology, Glasgow Royal Infirmary, Glasgow, Scotland, UK; ^83^ Department of Pathology, The University of Melbourne, Melbourne, Australia; ^84^ Department of Obstetrics, Gynecology and Oncology, Warsaw Medical University and Brodnowski Hospital, Warsaw, Poland; ^85^ Department of Health Science Research, Division of Biomedical Statistics and Informatics, Mayo Clinic, Rochester, Minnesota, USA; ^86^ Department of Obstetrics and Gynaecology, Affiliated with UM Cancer Research Institute, Faculty of Medicine, University of Malaya, Malaysia; ^87^ Department of Obstetrics and Gynecology, Duke University Medical Center, Durham, North Carolina, USA; ^88^ Cancer Division, QIMR Berghofer Medical Research Institute, Brisbane, Australia; ^89^ Department of Laboratory Medicine and Pathology, Mayo Clinic, Rochester, Minnesota, USA; ^90^ School of Public Health, The University of Texas, Houston, Texas, USA; ^91^ Department of Health Science Research, Division of Epidemiology, Mayo Clinic, Rochester, Minnesota, USA; ^92^ On behalf of the Australian Ovarian Cancer Study Group

**Keywords:** ovarian cancer, immunosuppression, biomarkers, genetic variation, TGFBR2

## Abstract

**Background:**

Regulatory T (Treg) cells, a subset of CD4+ T lymphocytes, are mediators of immunosuppression in cancer, and, thus, variants in genes encoding Treg cell immune molecules could be associated with ovarian cancer.

**Methods:**

In a population of 15,596 epithelial ovarian cancer (EOC) cases and 23,236 controls, we measured genetic associations of 1,351 SNPs in Treg cell pathway genes with odds of ovarian cancer and tested pathway and gene-level associations, overall and by histotype, for the 25 genes, using the admixture likelihood (AML) method. The most significant single SNP associations were tested for correlation with expression levels in 44 ovarian cancer patients.

**Results:**

The most significant global associations for all genes in the pathway were seen in endometrioid (*p* = 0.082) and clear cell (*p* = 0.083), with the most significant gene level association seen with (*p* = 0.001) and clear cell EOC. Gene associations with histotypes at< 0.05 included:(*p* = 0.005 and = 0.008, serous and high-grade serous, respectively), (*p* = 0.035, endometrioid and mucinous), (*p* = 0.03, mucinous), (*p* = 0.022, clear cell), (*p* = 0.021 endometrioid) and (*p* = 0.017 and = 0.025, endometrioid and mucinous, respectively).

**Conclusions:**

Common inherited gene variation in Treg cell pathways shows some evidence of germline genetic contribution to odds of EOC that varies by histologic subtype and may be associated with mRNA expression of immune-complex receptor in EOC patients.

## INTRODUCTION

Ovarian cancer is the leading cause of death due to gynecological cancers in the United States [1]. Although two-thirds of ovarian cancer patients initially respond to surgical debulking and chemotherapy [2], a majority eventually relapse [3, 4]. The five-year survival rate of ovarian cancer varies significantly across clinical stages, with almost 90% of stage I patients surviving, to just a little over 20% of advanced-stage patients surviving [5].

In recent years, host tumor immunosuppression has attracted research in ovarian cancer in hopes of identifying underlying biological mechanisms that determine the development and progression of ovarian cancer. Ovarian tumors have been found to induce migration of immunosuppressive cells into tumor tissue [6]. Thus, exploring molecular pathways underlying suppression of immune responses in ovarian cancer to identify novel targets for immunotherapy and/or to identify markers that can predict the risk of ovarian cancer may be a route to both treating this deadly disease and/or earlier identification.

An important pathway to consider in immune function is suppression of host immune response by regulatory T (Treg) cells, a subset of CD4+ T cells that maintain immune tolerance and inhibit the development of an antitumor immune response. In fact, higher prevalence of Treg cells has been found in various cancers [7–12], including ovarian cancer [13–16], compared to controls. Treg cells have been detected in ovarian tumors [15], as well as in malignant ascites [13] and peripheral blood [16] of ovarian cancer patients. Further, an association of ovarian cancer outcomes with genetic variation in Treg-related genes specific to induction, trafficking, or immunosuppressive function of Treg cells, also suggests a role for the Treg cell phenotype in ovarian cancer [17]. Given the importance of inherited factors in both ovarian cancer and Treg cells, we sought to characterize their role in ovarian cancer etiology. We conducted a comprehensive epidemiological study in which we investigated the significance of single nucleotide polymorphisms (SNPs) in the Treg cell pathway and mRNA expression profiles in epithelial ovarian cancer (EOC) etiology.

## RESULTS

The descriptive characteristics of the study population are presented in Table 1. The majority of EOC patients (*n* = 9,330) were of the serous histology. Compared to controls, cases were significantly older and more likely to report a family history of breast or ovarian cancer and a personal history of endometriosis. Conversely, pregnancy, tubal ligation, breastfeeding, and use of oral contraceptives (OCs) were more likely to be reported by controls.

**Table 1:** Descriptive characteristics of 15,596 ovarian cancer cases and 23,236 controls from the Ovarian Cancer Association Consortium (OCAC)

Variable	Case *N* = 15596	Control *N* = 23236	*P* value
**Age**^1^	57.36 (11.70)	55.61 (11.90)	<0.0001
**Ethnicity**^2^			
Non-Hispanic	13847 (99.6)	21539 (99.7)	0.03
Hispanic	56 (.4)	59 (.3)	
*Missing*	1693	1638	
**Family history of ovarian cancer**^2^			
No	5891 (91.6)	7643 (95.7)	<0.0001
Yes	543 (8.4)	343 (4.3)	
*Missing*	9162	15250	
**Height**^1^	1.64 (0.07)	1.63 (0.06)	<0.0001
*Missing*	4571	6596	
Weight^1^	57.2 (9.80)	56.4 (8.71)	
*Missing*	6600	9277	<0.0001
**Body Mass Index (BMI)**^1^	21.29 (3.43)	21.18 (3.06)	0.01
*Missing*	6642	9311	
Age at menarche^1^	12.8 (1.60)	12.9 (1.68)	0.02
*Missing*	4914	7195	
**Total number of pregnancies**^1^	2.37(1.80)	2.63(1.74)	<0.0001
*Missing*	4879	7066	
**Breast feeding**^2^			
No	3070 (40.5)	4078 (30.2)	<0.0001
Yes	4502 (59.5)	9426 (69.8)	
*Missing*	8024	9732	
**Menopausal status**^2^			
Pre/perimenopausal	3585 (32.4)	4519 (28)	<0.0001
Post-menopausal	7491 (67.6)	11640 (72.0)	
*Missing*	4520	7077	
**HRT**^2^			
No	2675 (44.3)	3237 (44.6)	0.73
Yes	3366 (55.7)	4025 (55.4)	
*Missing*	9555	15974	
**OC use**^2^			
Never	4465 (41.9)	6054 (37.4)	<0.0001
Ever	6191 (58.1)	10152 (62.6)	
*Missing*	4940	7030	
**OC use in months**^1^	38.21 (59.83)	49.40(69.27)	<0.0001
*Missing*	5164	7209	
**Tubal ligation**^2^			
No	8420 (84.4)	8278 (76.7)	<0.0001
Yes	1562 (15.7)	2514 (23.3)	
*Missing*	5614	12444	
**Endometriosis**^2^			
No	7435 (90.8)	10030 (93.2)	<0.0001
Yes	755 (9.2)	731 (6.8)	
*Missing*	7406	12475	
**Hysterectomy**^2^			
No	7352 (68.3)	13103 (81.2)	<0.0001
Yes	3413 (31.7)	3025 (18.8)	
*Missing*	4831	7108	
**Clinical characteristics Histology**^2^			
Serous	9330 (59.8)		
Mucinous	1592 (10.2)		
Endometrioid	2099 (13.5)		
Clear cell	1033 (6.6)		
Mixed Cell	505 (3.2)		
Other	1037 (6.7)		
**Behavior**^2^			
LMP	1724 (11.1)		
Invasive	13872 (88.9)		
**FIGO stage**^2^			
1	3488 (31.7)		
2	1147 (10.4)		
3	5412 (49.2)		
4	954 (8.7)		
**Grade**^2^			
Well differentiated	1240 (12.5)		
Moderately differentiated	2427 (24.4)		
Poorly differentiated	5591 (56.2)		
Undifferentiated	699 (7.0)		
*Missing*	*5639*		

1 Mean (standard deviation),

2 N(%), CI = Confidence interval, BMI = body mass index, HRT = hormone replacement therapy, OC = oral contraceptive, LMP = low malignant potential

### Association of genetic variation by histotype

*P*-values for the gene burden test for each gene in the pathway and the Treg cell pathway (all SNPs analyzed together) by histotype (serous, high-grade serous, endometrioid, clear cell, invasive mucinous) are presented in Table 2. The most significant burden test (*p* = 0.001) was seen with *TGFBR2* and clear cell EOC. Other gene associations with histotypes at *p* < 0.05 included: *IL12B* (*p* = 0.005 and *p* = 0.008, serous and high-grade serous, respectively), *IL8RA* (*p* = 0.035, endometrioid and invasive mucinous), *LGALS1* (*p* = 0.03, invasive mucinous), *STAT5B* (*p* = 0.022, clear cell), *TGFBR1* (*p* = 0.021, endometrioid) and TGFBR2 (*p* = 0.017 and *p* = 0.025, endometrioid and invasive mucinous, respectively). The most significant global associations for all genes in the Treg cell pathway were seen in endometrioid (*p* = 0.082) and clear cell (*p* = 0.083) EOC.

**Table 2:** Admixture maximum likelihood gene burden p-values for each gene in the Treg cell pathway and overall considering all genes

Gene	Serous (*n* = 9,330)	High-grade serous (*n* = 5,792)	Endometrioid (*n* = 2,060)	Clear cell (*n* = 1,021)	Invasive Mucinous (*n* = 933)
*CTLA4*	0.612	0.984	0.337	0.471	0.178
*FCRL3*	0.426	0.388	0.464	0.546	0.110
*FOXP3*	0.362	0.254	0.630	0.525	0.287
*GZMB*	0.484	0.203	0.220	0.931	0.847
*HDAC9*	0.679	0.864	0.212	0.398	0.990
*IL12B*	0.005	0.008	0.127	0.915	0.088
*IL17RA*	0.269	0.243	0.974	0.831	0.652
*IL23A*	0.137	0.111	0.990	0.431	0.561
*IL23R*	0.423	0.903	0.470	0.101	0.221
*IL2RA*	0.948	0.960	0.153	0.281	0.148
*IL7*	0.915	0.933	0.339	0.822	0.670
*IL7R*	0.558	0.562	0.296	0.459	0.670
*IL8RA*	0.118	0.084	0.035	0.344	0.035
*LGALS1*	0.222	0.054	0.841	0.520	0.030
*LGALS9*	0.958	0.949	0.649	0.885	0.081
*PRKCQ*	0.511	0.862	0.879	0.528	0.729
*STAT5A*	0.283	0.463	0.556	0.117	0.442
*STAT5B*	0.721	0.873	0.412	0.022	0.297
*TGFB1*	0.864	0.908	0.864	0.966	0.168
*TGFB2*	0.739	0.418	0.481	0.087	0.672
*TGFB3*	0.335	0.250	0.139	0.354	0.438
*TGFBR1*	0.378	0.398	0.021	0.504	0.493
*TGFBR2*	0.644	0.242	0.017	0.001	0.025
*TGFBR3*	0.068	0.256	0.446	0.295	0.366
*TNFSF14*	0.742	0.521	0.964	0.981	0.848
Treg cell gene pathway	0.444	0.719	0.082	0.083	0.632

Single SNP associations for each gene are shown in Supplemental Table 1. The effective number of independent SNPs tested was 370; applying a bonferroni correction for testing 370 SNPs across 5 groups, yields *p* < 2.7 × 10^−5^ as the significance threshold. No single SNPs remains significant after correction for multiple testing within histotype. The most single SNP association was seen with *TGFBR2* and clear cell; the T allele in rs3773636 was associated with a 21% increased risk of clear cell ovarian cancer (OR = 1.21, 95% CI = 1.10-1.33, *p* = 0.0001).

### eQTL in TGFBR2 associate with FCGR2B expression

**Figure 1:**
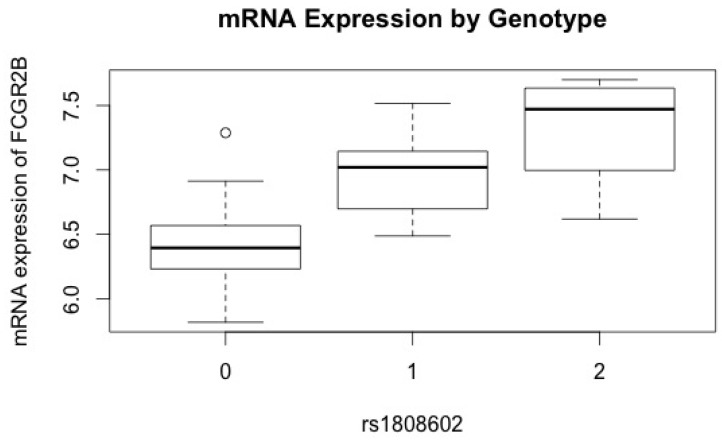
Association of variant alleles in *TGFBR2* with circulating mRNA expression levels in *FCGR2B* *FCGR2B* mRNA expression levels (y-axis) versus rs1808602 (x-axis). Each additional copy of the variant allele (G) in rs1808602 was associated with a significant increase in mRNA expression level after adjusting for age and histology.

*TGFBR2* contained the SNP with the most significant association with risk of clear cell EOC and also contained several additional SNPs with suggestive associations with clear cell and mucinous EOC. Thus, SNPs in *TGFBR2* were correlated with mRNA expression levels as measured by the 9,634 probes passing quality control (QC) and showing expression above the background in at least 25% of the samples [18]. Regression analyses showed the most significant association between rs1808602 and *FCGR2B* (*P_FDR_* < .05) with an adjusted r^2^ = 0.51 for a model including both SNP and histology; the variation attributable to the SNP alone was r^2^ = 0.45. Each additional copy of the minor (G) allele (minor allele frequency (MAF) = 42.4%) was associated with an increase in mRNA expression level of 0.51 in *FCGR2B* (Figure 1). This SNP-gene association was the only association significant after correction for multiple testing.

## DISCUSSION

Treg cells have been shown to suppress tumor antigen specific immunity in ovarian cancer, *in vitro* and *in vivo* [13]. However, the role of Treg cells in the etiology of ovarian cancer is not well established. We attempted to evaluate robust genetic biomarkers associated with Treg cells in relation to EOC in a large sample pooled from the Ovarian Cancer Association Consortium (OCAC). We hypothesized that SNPs in genes that regulate the function of Treg cells could potentially be associated with variation in immune response to ovarian tumors. Hence, in this study we evaluated SNPs in 25 genes thought to govern the function of Treg cells to determine their association to EOC. We found a modest association between *TGFBR2* and invasive clear cell EOC. SNPs in this gene have been found to be associated with other pathological conditions, including gastric and colorectal cancer [19, 20]. The TGF-β family of cytokines plays an important role in proliferation, differentiation, and apoptosis of many cell types [21]. However, some tumors, such as ovarian tumors, evade the anti-proliferative effects of TGF-β by acquiring mutations in TGF-β signaling pathway [22]. Furthermore, the TGF-β signaling pathway plays a paradoxical role in tumorigenesis, initially suppressing and later promoting tumor growth and metastasis [23].

The significant association of rs1808602 in *TGFBR2* with lymphoblastoid cell line (LCL) mRNA expression of *FCGR2B* (FcγRIIB) adds evidence for an immune component in ovarian carcinogenesis. *FCGR2B* binds to the Fc component of the antigen-IgG immune complex, suppressing immune response through several mechanisms, including inhibition of antigen presentation to T lymphocytes as well as reduced phagocytosis by neutrophils [24]. The only inhibitory receptor among members of the FcGR family in humans, *FCGR2B*, expressed on B lymphocytes [25] and follicular dendritic cells, is thought to be critical for maintenance of humoral immune response [26, 27]. The modest correlation between the *TGFBR2* polymorphism and mRNA expression of *FCGR2B* observed suggests that TGF-β cytokine signaling pathway may, directly or indirectly through Treg cells, regulate the expression of FcGR, thereby potentially altering the balance between pro-inflammatory and anti-inflammatory immune response. Furthermore, the downstream inhibitory effect of *FCGR2B* expression is not limited to immune cells. Experimental models have demonstrated the potential of *FCGR2B* to promote tumorigenesis when expressed on non-lymphoid tumor cells [28, 29]. *FCGR2B* expression is thought to be a mechanism of immune escape by tumor cells [30]. Thus, our findings indicate that polymorphisms in *TGFBR2* may potentially affect inter-individual variation in anti-tumor immune response through FcG receptor modulation. Additional evidence for Treg-cell-related eQTL SNPs has been seen with survival in ovarian cancer [31, 32]. Specifically, genetic variation in *CD80* was associated with poorer survival of endometrioid cases and with increased tumor *CD80* expression. The above findings suggest that inherited factors contributing to ovarian cancer etiology and outcome may, in part, drive the expression of important immune-related genes.

**Figure 2:**
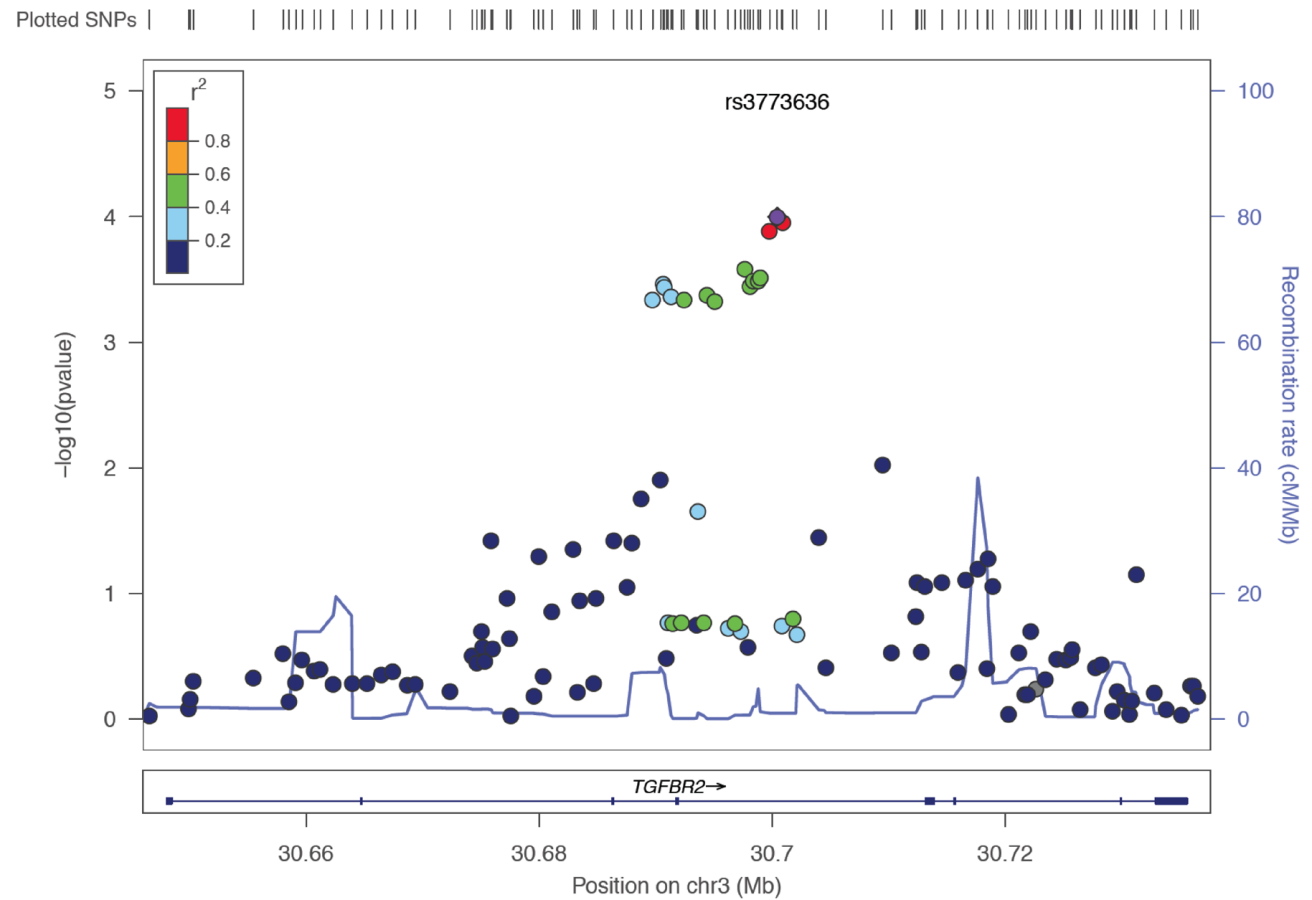
Linkage disequilibrium structure and regional association map of *TGFBR2* with risk of clear cell ovarian cancer Each dot indicates a SNP, with the corresponding region on Chromosome 3 (x axis) and negative log10 p-value (y axis) associated with the SNP; color-coding reflects pairwise linkage disequilibrium. The purple dot is rs3773636, the most significant genetic association with clear cell ovarian cancer (*p* = 0.0001). It is located on Chromosome 3 at 30,690,658 bp (hg19) in *TGFBR2*.

Further evaluation of the structure of *TGFBR2* showed that the rs3773636 SNP is in strong linkage disequilibrium (r^2^ = 1) with a SNP (rs995435) that is thought to likely affect binding of proteins such as *HNF4A, EP300,* and *GATA2*, all associated with the balance of cell differentiation [33] (Figure 2). This SNP resides in *SMAD4* and *ELF5* (an ETS-related transcription factor) motifs in a relatively important position. In addition, we find that rs1463535 in *TGFBR2*, ~2 Mb from rs3773636 and independent of rs3773636, is associated (*p* < 8e-05) with expression of *TGFBR2* in lymphoblastoid cell lines (*p* < 8e-05) [34].

Although we find relatively weak associations between SNPs in the Treg cell pathway and EOC etiology, we do see modest evidence that *TGFBR2* contains an eQTL that is perhaps modulating expression of inhibitory immune-complex receptor genes. Thus, the Treg cell genetic hypothesis perhaps merits further investigation in a larger, more diverse population.

## MATERIALS AND METHODS

### SNP selection

An extensive literature review of studies examining the role of regulatory T cells in immune response was conducted in 2010, and genes relevant to the function of Treg cells were identified. Tag SNPs in 25 genes (MAF ≥ 0.05),were selected using the SNP database on Genome Variation Server [35]. SNP selection parameters included an r^2^ > = 0.8 and the Centre d'Etude du Polymorphisme Humain (CEPH) reference population. The genomic region was expanded upstream and downstream (5 Kb) of each gene using linkage disequilibrium block structure to capture tag SNPs in regulatory regions. Tag SNPs were then assessed for design scores using Illumina's Assay Design Tool for Infinium, and SNPs with a design score < 0.4 were excluded. SNPs were also excluded if the call rate was < 95%, if the test for deviation from Hardy Weinberg equilibrium proportions in controls was *p* < 10^−4^, or if greater than 2% discordance in duplicate pairs was observed. Of the 1,358 SNPs from the Treg cell pathway that were included for genotyping, a total of 1,351 passed QC and were included in the analysis presented in this paper (Supplemental Table 2).

### Study population, genotyping, and quality control

Germline DNA (250 ng genomic or 750 ng whole-genome amplified) from a total of 15,596 ovarian cancer cases and 23,236 controls from 40 studies in the OCAC (Supplemental Table 3) was genotyped on a custom Illumina iSelect BeadArray. OCAC is an international, multidisciplinary consortium, comprising population-based, hospital-based and nested case-control, and case-only studies of ovarian cancer, conducted in the United States, Europe, Asia, and Australia. Genotype calling and quality control procedures were described previously [36, 37]. Samples with a genotype call rate of < 95% were excluded. Hap Map samples from European (CEU, *N* = 60), African (YRI, *N* = 53), and Asian (JPT+CHB, *N* = 88) populations were used to estimate intercontinental ancestry for each individual using the Local Ancestry in Admixed Population (LAMP) program [38], and variation in population substructure was estimated using principal components (PCs). Only individuals with a LAMP score greater than 90% European ancestry were included in the present analyses.

### Statistical analyses

Logistic regression analyses in PLINK were used to test for evidence of additive associations of SNPs by histotype and restricted to invasive tumor behavior [39]. Evaluation of the scree plot of eigenvectors, derived using Eigenstrat, revealed that five PCs explained most of the variation in population substructure; the logistic regression models were adjusted accordingly for PCs, along with age. PC analysis was done using an in-house program written in C++ using the Intel MKL libraries for eigenvectors (available at http://ccge.medschl.cam.ac.uk/software/) [40]. We used the approach of Li et al. to calculate the effective number of independent SNPs tested, and this value was then used in a Bonferroni correction to determine single SNP significance [41, 42]. Regional association plots for SNPs with significant associations were constructed using LocusZoom software [43].

Both gene-level tests of association and global Treg cell pathway analyses by ovarian cancer histotypes were conducted using the admixture likelihood (AML) method [40, 44]. The AML method assumes a proportion of variants in each gene or pathway (α) is associated with outcome. The effect size of each SNP is assumed to be on a non-central χ2 distribution with non-centrality parameter η, which approximately captures that SNP's contribution to the total genetic variance of the outcome. To accommodate the correlation between SNPs in each gene, AML uses a pseudo-maximum likelihood method to estimate the α and η. For each gene-level and pathway-level test, we performed 1,000 simulations, assuming that the maximum proportion of associated SNPs in each gene or pathway was 0.20. We report p-values for the AML trend test.

### Expression quantitative trait loci (eQTL) analysis in ovarian cancer patients

We measured *trans* and *cis* genotype associations with mRNA expression levels in LCL collected pre-treatment from unrelated EOC cases enrolled in the Gilda Radner Ovarian Family Cancer Registry (GRR) at Roswell Park Cancer Institute (RPCI), a part of the larger OCAC study described above. Microarray-based gene expression was assayed using the Illumina HumanHT-12v3 Gene Expression Beadchip, with almost 50,000 probes derived from the National Center for Biotechnology Information Reference Sequence (NCBI) RefSeq (Build 36.2, Rel 22) and the UniGene (Build 199) databases [45]. Beadscan was used to scan and extract the raw intensity and the data corrected by local background subtraction in GenomeStudio module. A quantile normalization algorithm in the lumi package in the R-based Bioconductor Package was used to normalize the log_2_ transformed intensity data. For data QC, we excluded the probes with detection P value > 0.05 (the P values were generated in BeadStudio software) in at least 25% of the samples, yielding 9,634 genes (18). Both LCL mRNA levels and genotype data were available on 44 patients with EOC from the GRR. Genes containing the SNPs most significantly associated with risk of EOC were selected for SNP-mRNA expression level analyses using linear regression adjusted for patient age and histotype. All analyses were corrected for multiple testing [46].

## SUPPLEMENTARY MATERIAL


