# Supplementary material for: Assessment of variation in immunosuppressive pathway genes reveals TGFBR2 to be associated with risk of clear cell ovarian cancer
Source: Oncotarget. 2016 Jun 21;7(43):69097–110. doi: 10.18632/oncotarget.10215 (PMC5340115; doi:10.18632/oncotarget.10215)
Supplement: Supplementary file 1 [file oncotarget-07-69097-s001.pdf]

Assessment of variation in immunosuppressive pathway genes reveals TGFBR2 to be associated with risk of clear cell ovarian cancer

Supplementary Material

Supplemental Table 1. Most significant single SNP associations by gene for all SNPs in the Treg cell pathway

Gene	All Histotypes					Serous					Endometrioid					Clear cell					Invasive Mucinous				
	SNP	P	EAF	Eff/ Ref	OR	SNP	P	EAF	Eff/ Ref	OR	SNP	P	EAF	Eff/ Ref	OR	SNP	P	EAF	Eff/ Ref	OR	SNP	P	EAF	Eff/ Ref	OR
CTLA4	rs3087243	0.478	0.442	A/G	1.01	rs11571317	0.705	0.071	T/C	0.99	rs231777	0.134	0.851	C/T	0.94	rs3087243	0.215	0.442	A/G	1.06	rs3087243	0.253	0.442	A/G	0.95
FCRL3	rs1537947	0.033	0.214	C/G	0.96	rs11264793	0.041	0.237	T/A	0.96	rs6691569	0.104	0.270	A/G	1.06	rs6691569	0.228	0.270	A/G	1.06	rs11264798	0.056	0.504	G/C	1.09
FOXP3	rs2280883	0.334	0.437	C/T	1.01	rs2280883	0.274	0.437	C/T	1.02	rs3761549	0.719	0.125	A/G	0.98	rs3761549	0.537	0.125	A/G	0.96	rs3761549	0.176	0.125	A/G	1.09
GZMB	rs8192920	0.144	0.244	A/C	1.03	rs7144366	0.147	0.598	C/T	1.03	rs10873219	0.159	0.199	T/G	1.06	rs8192920	0.336	0.244	A/C	1.05	rs7144366	0.415	0.598	C/T	1.04
HDAC9	rs6973918	0.006	0.878	G/A	1.07	rs2190273	0.003	0.878	C/T	1.09	rs957958	0.001	0.389	G/A	0.89	rs7802855	0.008	0.396	C/T	0.88	rs12672843	0.033	0.047	C/G	1.28
IL12B	rs6894567	0.002	0.198	G/A	1.06	rs6894567	0.009	0.198	G/A	1.06	rs1003199	0.045	0.502	T/C	0.94	rs2569253	0.245	0.508	C/T	0.95	rs2546892	0.007	0.166	A/G	0.84
IL17RA	rs879574	0.057	0.112	A/T	1.04	rs13053889	0.013	0.196	T/C	0.95	rs13053889	0.173	0.196	T/C	1.06	rs2241044	0.317	0.516	C/A	1.05	rs5748863	0.168	0.655	A/G	0.93
IL23A	rs11171806	0.408	0.069	A/G	1.02	rs11171806	0.359	0.069	A/G	1.03	rs11171806	0.639	0.069	A/G	1.03	rs11171806	0.453	0.069	A/G	0.93	rs11171806	0.856	0.069	A/G	0.98
IL23R	rs10889664	0.024	0.337	T/C	1.04	rs10889675	0.017	0.112	A/C	1.07	rs12401432	0.057	0.299	G/C	1.07	rs4655686	0.032	0.300	A/T	1.11	rs1343151	0.010	0.343	A/G	0.88
IL2RA	rs2476491	0.080	0.302	T/A	0.97	rs2031229	0.149	0.229	A/G	0.97	rs7072398	0.020	0.472	A/G	0.93	rs11256456	0.015	0.210	C/T	1.14	rs12722527	0.056	0.160	T/C	1.12
IL7	rs17505589	0.097	0.098	T/C	1.04	rs17505589	0.036	0.098	T/C	1.06	rs2887501	0.095	0.099	C/A	1.09	rs2583759	0.336	0.189	C/T	0.94	rs3888020	0.149	0.380	A/C	1.07
IL7R	rs6891095	0.099	0.140	C/T	0.96	rs10053847	0.040	0.141	A/G	0.95	rs6891095	0.035	0.140	C/T	0.90	rs6891095	0.239	0.140	C/T	1.08	rs11567762	0.304	0.141	A/G	0.93
IL8RA	rs3138060	0.099	0.051	C/G	1.06	rs3138060	0.142	0.051	C/G	1.06	rs1567868	0.012	0.949	A/G	0.84	rs1008563	0.047	0.434	A/G	1.10	rs3138060	0.100	0.051	C/G	0.83
LGALS1	rs9622682	0.055	0.433	A/G	0.97	rs9622682	0.154	0.433	A/G	0.98	rs929039	0.773	0.339	C/T	0.99	rs4820294	0.786	0.339	A/G	1.01	rs9622682	0.023	0.433	A/G	0.90
LGALS9	rs3763959	0.345	0.567	G/A	0.99	rs4239242	0.270	0.363	C/T	1.02	rs3763959	0.107	0.567	G/A	0.95	rs4794976	0.667	0.308	G/T	0.98	rs4239242	0.023	0.363	C/T	0.90
PRKCQ	rs6602820	0.059	0.570	A/G	1.03	rs4750517	0.057	0.318	G/A	1.04	rs943452	0.082	0.378	T/C	1.06	rs11259403	0.069	0.665	T/C	0.92	rs2026431	0.012	0.299	T/G	1.13
STAT5A	rs9906989	0.039	0.170	T/G	0.96	rs9906989	0.061	0.170	T/G	0.96	rs2293158	0.251	0.283	C/T	0.96	rs16967637	0.044	0.283	A/C	1.10	rs7217728	0.117	0.285	C/T	0.92
STAT5B	rs17591972	0.180	0.086	G/A	0.96	rs17591972	0.252	0.086	G/A	0.96	rs9907247	0.220	0.288	A/G	0.96	rs16967611	0.050	0.291	G/A	1.10	rs8064638	0.083	0.290	A/G	0.92
TGFB1	rs8179181	0.268	0.230	A/G	0.98	rs8179181	0.312	0.230	A/G	0.98	rs8110090	0.162	0.054	G/A	0.90	rs8179181	0.321	0.230	A/G	1.05	rs8179181	0.044	0.230	A/G	0.90
TGFB2	rs4335431	0.019	0.077	C/T	0.94	rs2027566	0.028	0.676	A/C	0.96	rs12029576	0.121	0.708	A/C	1.06	rs1417488	0.004	0.250	T/C	0.85	rs2799086	0.065	0.134	T/C	0.88
TGFB3	rs3917148	0.239	0.078	G/T	1.03	rs3917148	0.072	0.078	G/T	1.06	rs11466414	0.089	0.057	A/G	0.89	rs3917158	0.165	0.833	C/T	1.09	rs3917148	0.304	0.078	G/T	0.91
TGFBR1	rs10512263	0.044	0.069	C/T	1.06	rs10512263	0.016	0.069	C/T	1.08	rs10733710	0.042	0.221	A/G	0.92	rs928180	0.118	0.090	G/A	0.88	rs928180	0.008	0.090	G/A	1.22
TGFBR2	rs11466515	0.196	0.309	C/A	0.98	rs3773645	0.106	0.313	G/C	0.97	rs12493607	0.001	0.340	C/G	0.89	rs3773636	.0001	0.260	T/C	1.21	rs9790268	0.002	0.453	T/C	1.16
TGFBR3	rs2029356	0.025	0.746	C/T	0.96	rs12089918	0.012	0.216	G/C	0.95	rs6678564	0.014	0.088	C/G	0.86	rs284172	0.013	0.846	T/A	0.86	rs1805117	0.019	0.175	C/T	1.15
TNFSF14	rs12609318	0.331	0.248	A/G	0.98	rs12609318	0.386	0.248	A/G	0.98	rs11878563	0.343	0.571	G/A	0.97	rs8106574	0.439	0.770	C/T	1.04	rs1077667	0.474	0.211	T/C	1.04

SNP= most significantly associated SNP in gene; p=p-value from single SNP test of association; EAF=Effect allele frequency; Eff/Ref=Effect allele/Reference allele; OR=Odds ratio for each additional copy of the effect allele

Supplemental Table 2. Tag SNPs per gene in Treg cell pathway (n=1351)

Gene	Tag SNPs	Gene	Tag SNPs
CTLA4	5	*LGALS1*	3
FCRL3	14	*LGALS9*	11
FOXP3	4	*PRKCQ*	145
GZMB	9	*STAT5A*	7
HDAC9	418	*STAT5B*	16
IL12B	21	*TGFB1*	6
IL17RA	20	*TGFB2*	55
IL23A	1	*TGFB3*	18
IL23R	58	*TGFBR1*	37
IL2RA	55	*TGFBR2*	110
IL7	38	*TGFBR3*	255
IL7R	34	*TNFSF14*	4
IL8RA	7		

Supplemental Table 3. Frequency distribution of controls and cases by histologic subtypes in 40 studies participating in the Ovarian Cancer Association Consortium

			Histological subtypes						
Study	Control N %	All case N %	Serous N %	Mucinous N %	Endometrioid N %	Clear N %	Mixed Cell N %	Other N %	Total
AUS	978 (4.21)	848 (5.44)	561 (6.01)	38 (2.39)	112 (5.34)	52 (5.03)	60 (11.88)	25 (2.41)	848
BAV	143 (0.62)	97 (0.62)	60 (0.64)	9 (0.57)	13 (0.62)	6 (0.58)	0 (0)	9 (0.87)	97
BEL	1347 (5.8)	272 (1.74)	194 (2.08)	23 (1.44)	22 (1.05)	23 (2.23)	8 (1.58)	2 (0.19)	272
DOV	1487 (6.4)	1084 (6.95)	710 (7.61)	149 (9.36)	160 (7.62)	65 (6.29)	0 (0)	0 (0)	1084
GER	413 (1.78)	209 (1.34)	108 (1.16)	29 (1.82)	21 (1)	6 (0.58)	2 (0.4)	43 (4.15)	209
GRR	0 (0)	106 (0.68)	72 (0.77)	5 (0.31)	18 (0.86)	10 (0.97)	1 (0.2)	0 0	106
HAW	157 (0.68)	80 (0.51)	46 (0.49)	15 (0.94)	12 (0.57)	5 (0.48)	2 (0.4)	0 (0)	80

HJO	273	245	146	14	26	4	11	44	245
	(1.17)	(1.57)	(1.56)	(0.88)	(1.24)	(0.39)	(2.18)	(4.24)	
HMO	138	142	50	7	12	1	0	72	142
	(0.59)	(0.91)	(0.54)	(0.44)	(0.57)	(0.1)	(0)	(6.94)	
HOC	447	224	120	44	29	13	0	18	224
	(1.92)	(1.44)	(1.29)	(2.76)	(1.38)	(1.26)	(0)	(1.74)	
HOP	1466	678	416	52	85	44	68	13	678
	(6.31)	(4.35)	(4.46)	(3.27)	(4.05)	(4.26)	(13.47)	(1.25)	
HSK	0	150	116	1	16	0	2	15	150
	0	0.96	1.24	0.06	0.76	0	0.4	1.45	
LAX	0	257	217	12	18	9	1	0	257
	(0)	(1.65)	(2.33)	(0.75)	(0.86)	(0.87)	(0.2)	(0)	
MAL	828	568	331	109	56	33	0	39	568
	(3.56)	(3.64)	(3.55)	(6.85)	(2.67)	(3.19)	(0)	(3.76)	
MAY	743	777	552	41	98	32	41	13	777
	(3.2)	(4.98)	(5.92)	(2.58)	(4.67)	(3.1)	(8.12)	(1.25)	
MCC	69	57	34	7	7	6	2	1	57
	(0.3)	(0.37)	(0.36)	(0.44)	(0.33)	(0.58)	(0.4)	(0.1)	
MDA	384	312	190	27	28	4	57	6	312

	(1.65)	(2)	(2.04)	(1.7)	(1.33)	(0.39)	(11.29)	(0.58)	
MSK	593<br>(2.55)	450<br>(2.89)	367<br>(3.93)	0<br>(0)	19<br>(0.91)	18<br>(1.74)	26<br>(5.15)	20<br>(1.93)	450
NCO	792<br>(3.41)	799<br>(5.12)	524<br>(5.62)	83<br>(5.21)	113<br>(5.38)	79<br>(7.65)	0<br>(0)	0<br>(0)	799
NEC	1009<br>(4.34)	904<br>(5.8)	521<br>(5.58)	112<br>(7.04)	130<br>(6.19)	102<br>(9.87)	19<br>(3.76)	20<br>(1.93)	904
NHS	425<br>(1.83)	134<br>(0.86)	78<br>(0.84)	17<br>(1.07)	14<br>(0.67)	6<br>(0.58)	5<br>(0.99)	14<br>(1.35)	134
NJO	181<br>(0.78)	169<br>(1.08)	100<br>(1.07)	7<br>(0.44)	27<br>(1.29)	20<br>(1.94)	7<br>(1.39)	8<br>(0.77)	169
NOR	371<br>(1.6)	246<br>(1.58)	139<br>(1.49)	18<br>(1.13)	27<br>(1.29)	11<br>(1.06)	17<br>(3.37)	34<br>(3.28)	246
NTH	323<br>(1.39)	248<br>(1.59)	117<br>(1.25)	34<br>(2.14)	64<br>(3.05)	20<br>(1.94)	3<br>(0.59)	10<br>(0.96)	248
ORE	0<br>(0)	65<br>(0.42)	48<br>(0.51)	4<br>(0.25)	4<br>(0.19)	2<br>(0.19)	5<br>(0.99)	2<br>(0.19)	65
OVA	748<br>(3.22)	764<br>(4.9)	427<br>(4.58)	95<br>(5.97)	105<br>(5)	57<br>(5.52)	8<br>(1.58)	72<br>(6.94)	764

POC	417<br>(1.79)	362<br>(2.32)	200<br>(2.14)	33<br>(2.07)	39<br>(1.86)	9<br>(0.87)	0<br>(0)	81<br>(7.81)	362
POL	223<br>(0.96)	211<br>(1.35)	106<br>(1.14	17<br>(1.07)	37<br>(1.76)	10<br>(0.97)	41<br>(8.12)	0<br>(0)	211
PVD	0<br>(0)	163<br>(1.05)	128<br>(1.37)	10<br>(0.63)	14<br>(0.67)	8<br>(0.77)	0<br>(0)	3<br>(0.29)	163
RMH	0<br>(0)	144<br>(0.92)	49<br>(0.53)	16<br>(1.01)	27<br>(1.29)	17<br>(1.65)	9<br>(1.78)	26<br>(2.51)	144
SEA	5770<br>(24.83)	1464<br>(9.39)	605<br>(6.48)	193<br>(12.12)	228<br>(10.86)	145<br>(14.04)	44<br>(8.71)	249<br>(24.01)	1464
SOC	0<br>(0)	274<br>(1.76)	108<br>(1.16)	46<br>(2.89)	62<br>(2.95)	11<br>(1.06)	13<br>(2.57)	34<br>(3.28)	274
SRO	0<br>(0)	132<br>(0.85)	92<br>(0.99)	3<br>(0.19)	17<br>(0.81)	8<br>(0.77)	2<br>(0.4)	10<br>(0.96)	132
STA	349<br>(1.5)	267<br>(1.71)	160<br>(1.71)	19<br>(1.19)	41<br>(1.95)	21<br>(2.03)	8<br>(1.58)	18<br>(1.74)	267
TOR	440<br>(1.89)	557<br>(3.57)	339<br>(3.63)	39<br>(2.45)	132<br>(6.29)	34<br>(3.29)	5<br>(0.99)	8<br>(0.77)	557

UCI	367 (1.58)	415 (2.66)	252 (2.7)	73 (4.59)	48 (2.29)	23 (2.23)	0 (0)	19 (1.83)	415
UKO	1104 (4.75)	650 (4.17)	353 (3.78)	75 (4.71)	113 (5.38)	65 (6.29)	26 (5.15)	18 (1.74)	650
UKR	0 (0)	47 (0.3)	23 (0.25)	3 (0.19)	6 (0.29)	2 (0.19)	1 (0.2)	12 (1.16)	47
USC	1047 (4.51)	822 (5.27)	539 (5.78)	103 (6.47)	79 (3.76)	35 (3.39)	0 (0)	66 (6.36)	822
WOC	204 (0.88)	203 (1.3)	132 (1.41)	10 (0.63)	20 (0.95)	17 (1.65)	11 (2.18)	13 (1.25)	203
**Total**	**23236**	**15596**	**9330**	**1592**	**2099**	**1033**	**505**	**1037**	**15596**

Note: Additional SNPs lying within 30,000 bp upstream and downstream of Treg related genes are not included.
